# Supplementary material for: AAV-Mediated Gene Delivery to 3D Retinal Organoids Derived from Human Induced Pluripotent Stem Cells
Source: Int J Mol Sci. 2020 Feb 3;21(3):994. doi: 10.3390/ijms21030994 (PMC7036814; doi:10.3390/ijms21030994)
Supplement: Supplementary file 1 [file ijms-21-00994-s001.pdf]

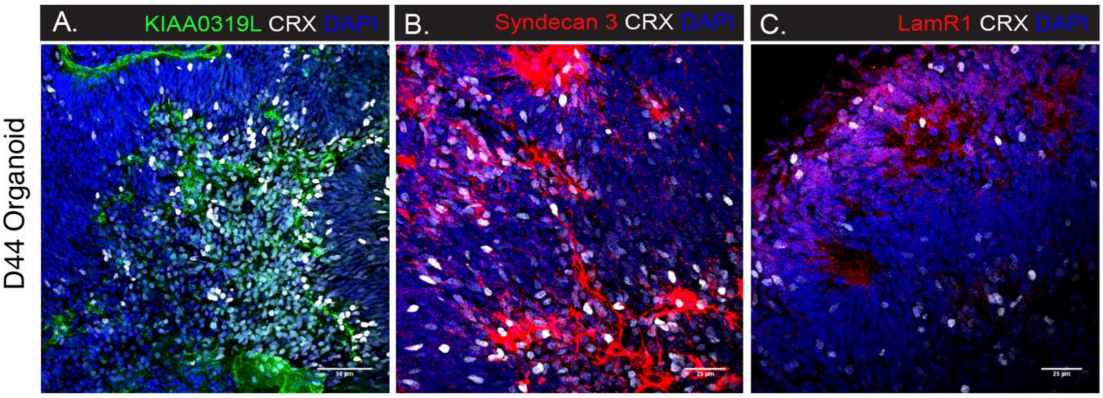

Figure S1. Colocalization of AAV binding elements and the photoreceptor-specific transcription factor CRX within retinal organoids at the day of infection. Expression of CRX in cells also expressing AAVR KIAA0319L (A.), Syndecan 3 (B.) and Laminin Receptor 1 (C.) in day 44 old hiPSC-derived organoids corresponding to photoreceptor cells

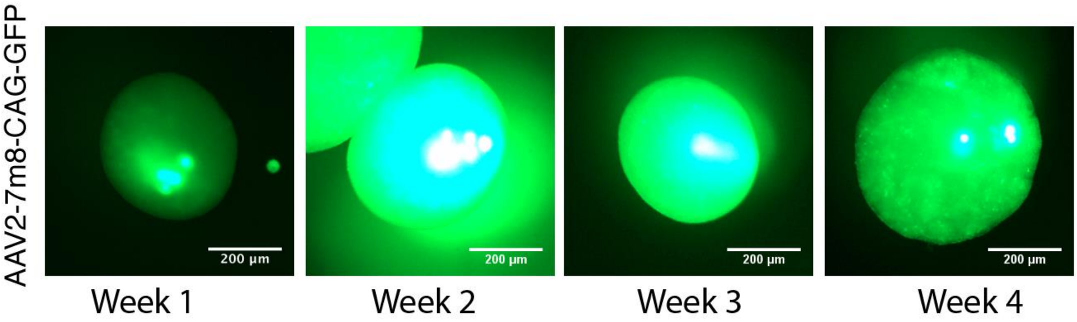

Figure S2. Kinetics of AAV2-7m8-driven transgene expression in human retinal organoids. Macroscopic analysis of live GFP expression in intact organoids in a 4-weeks follow-up.

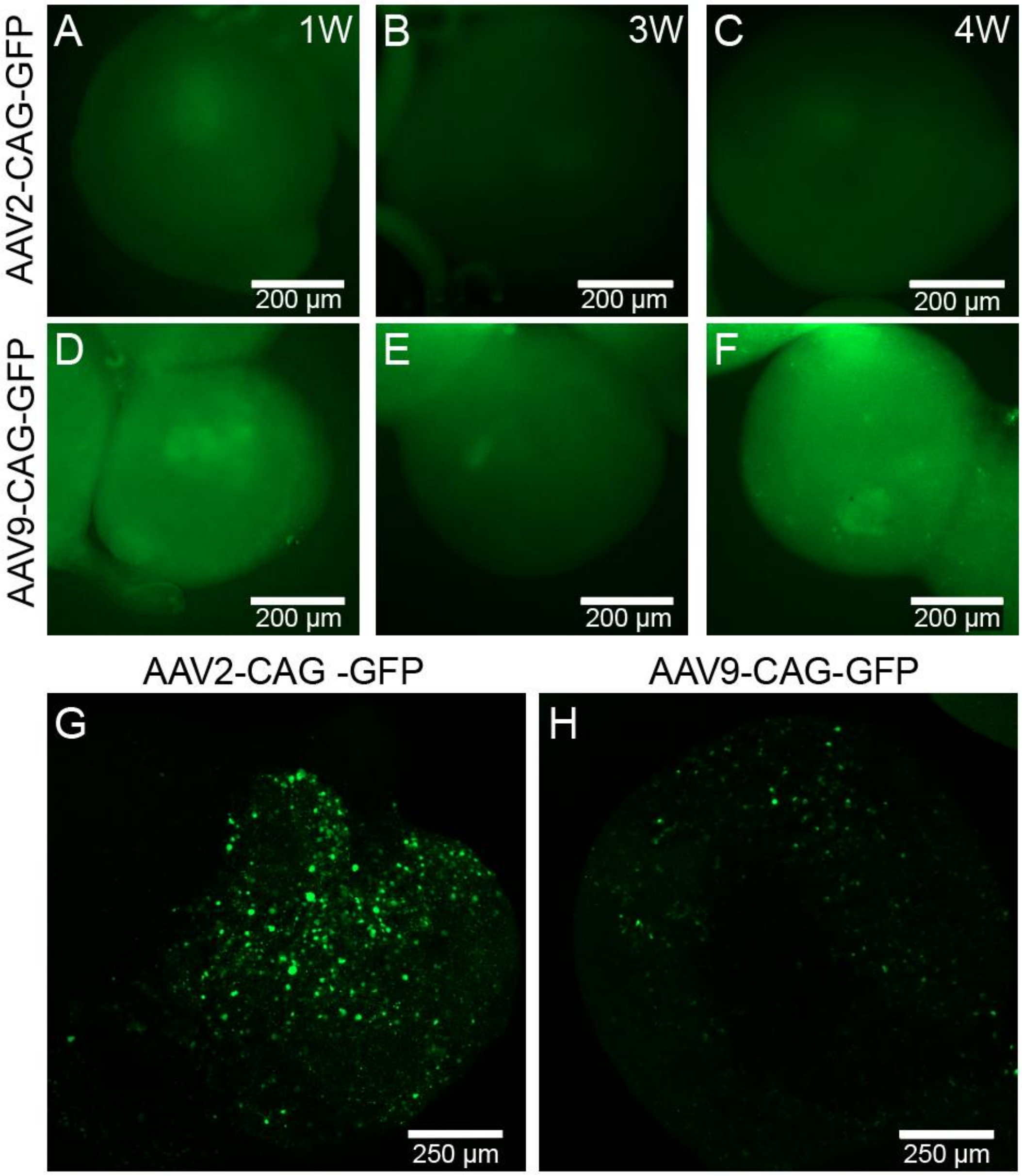

Figure S3. Infections at a dose of 5x1011vg per organoid for AAV2 (A-C) and AAV9 (D-F) as imaged on intact organoids using a macroscope. GFP expression-driven by AAV2 (G) and AAV9 (H) on cryosections of organoids imaged using confocal microscopy.

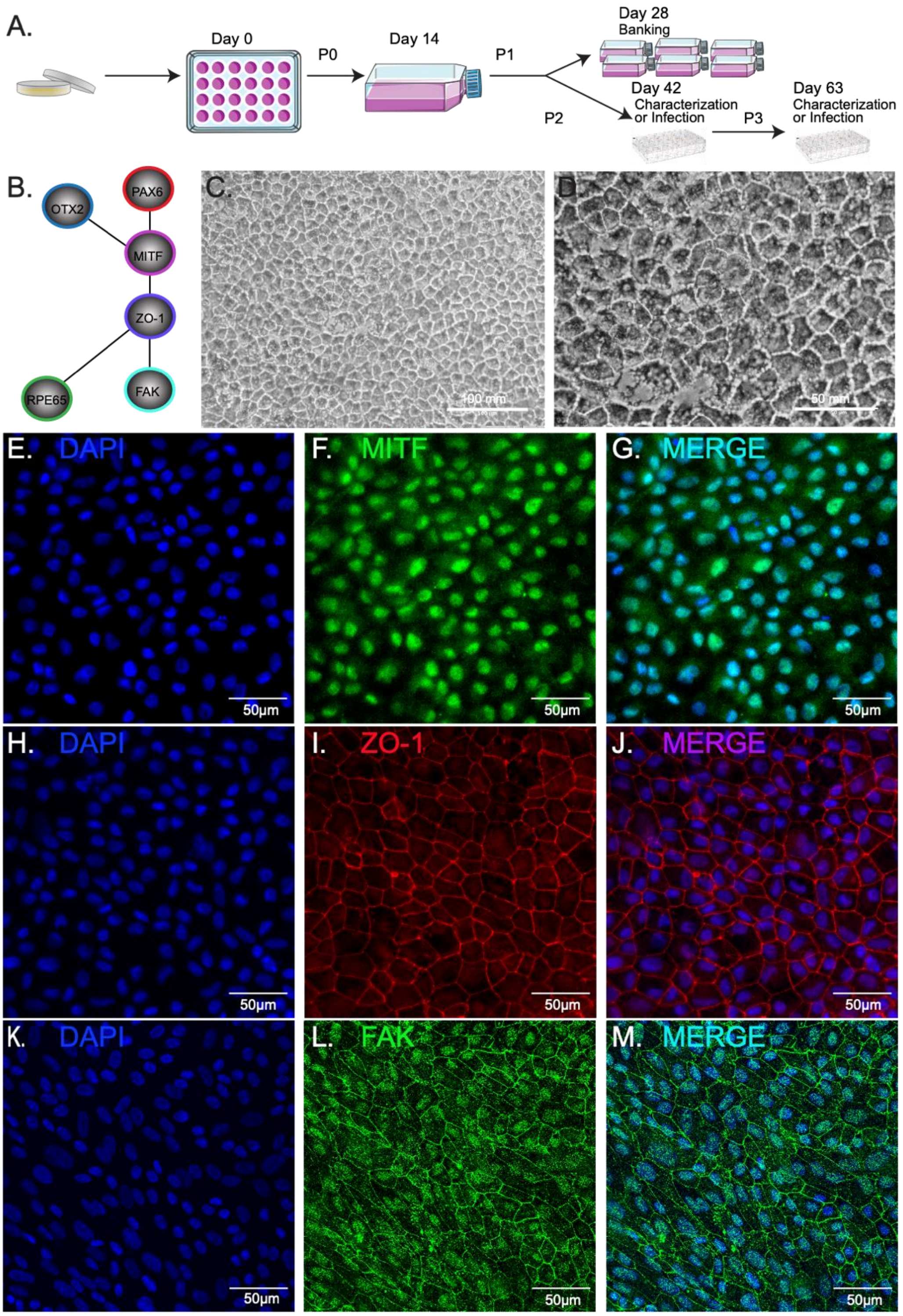

Figure S4. Characterization of hiRPE cells. A. Schematics of RPE generation steps, banking and time points for characterization or infections. **B.** Protein interactome network controlling the expression of genes responsible for the specification of the vertebrate RPE, analyzed using String and annotated data. OTX2 (Orthodenticle Homeobox 2) PAX6 (Paired Box 6), MITF (microphthalmia-associated transcription factor), ZO-1 (TJP1, tight junction protein 1), FAK (PTK2, protein tyrosine kinase 2) and RPE65 (Retinal pigment epithelium-specific protein 65kDa) [31]. **C-D.** Bright-field images of human RPE cultures at two different passages. **E-M** Immunofluorescence analysis of RPE specific markers in hiPSC-derived RPE cells. Scale bars: C: 100 µm; D-M: 50 µm
